# Holding time has limited impact on constitutive innate immune function in a long-lived Antarctic seabird, the Adélie penguin: implications for field studies

**DOI:** 10.1242/bio.059512

**Published:** 2023-02-23

**Authors:** Olivia Hicks, Akiko Kato, Danuta M. Wisniewska, Coline Marciau, Frédéric Angelier, Yan Ropert-Coudert, Arne Hegemann

**Affiliations:** ^1^ Centre d'Etudes Biologiques de Chizé, CNRS, La Rochelle Université, UMR 7372, Villiers-en-Bois, France; ^2^ Department of Biology, Lund University, Ecology Building, 223 62 Lund, Sweden; ^3^ Department of Biology, University of Southern Denmark, 5230 Odense, Denmark

**Keywords:** Antarctic, Ecoimmunology, Field study, Stress

## Abstract

There is great interest in measuring immune function in wild animals. Yet, field conditions often have methodological challenges related to handling stress, which can alter physiology. Despite general consensus that immune function is influenced by handling stress, previous studies have provided equivocal results. Furthermore, few studies have focused on long-lived species, which may have different stress-immune trade-offs compared to short-lived species that have primarily been tested. Here, we investigate whether capture and handling duration impacts innate immune function in a long-lived seabird, the Adélie penguin (*Pygoscelis adeliae*). We found no evidence for changes in three commonly used parameters of innate immune function upon holding time of up to 2 h, suggesting that immune function in this species is more robust against handling than in other species. This opens up exciting possibilities for measuring immune function in species with similar life-histories even if samples cannot be taken directly after capture.

## Teaser

**Summary:** No evidence found for the impact of handling duration on constitutive innate immune function in breeding Adélie penguins.

## INTRODUCTION

With growing interest in revealing proximate mechanisms driving ecological and evolutionary patterns, many studies include measurements of physiological parameters. In vertebrates one prominent example is measurements of immune function (Martin et al., 2014; Norris and Evans, 2000; Ricklefs and Wikelski, 2002). In the fast growing field of eco-immunology, it is crucial to ensure that measurements are accurate and to avoid confounding (methodological) effects. This can be particularly challenging when working with free-living animals that can be affected by the sampling procedures (Matson et al., 2005). For most physiological parameters, including immune function, handling stress represents an important potential methodological issue (Buehler et al., 2008; D'Amico et al., 2017; Zylbergberg, 2015). Stress and immunity parameters are linked through complex interactions between the neuroendocrine and immune axes (McEwen et al., 1997), with stress responses suppressing some forms of immunity while enhancing others (D'Amico et al., 2017). For instance in birds and mammals, stress-induced increase in glucocorticoid levels generally occur within a few minutes of capture (Romero and Reed, 2005; Romero and Romero, 2002), and these hormonal changes can subsequently and rapidly impact multiple immune pathways (reviewed in Martin, 2009). Furthermore, these stress responses may vary between individuals and species and they can therefore blur the potential effects of individual or environmental factors on immunity.

Stress-induced immunomodulation of constitutive innate immune function commonly occurs due to the trade-off between resources that are necessary to sustain the activation and maintenance of the stress response and the immune system (Evans et al., 2015; Martin et al., 2008; Moore and Hopkins, 2009; Nebel et al., 2012). Therefore, this trade-off may change as a function of the duration of exposure to the stressor (Martin, 2009). Some immune parameters can be highly sensitive to handling stress, while others might not show changes for hours meaning some assays could be better suited to certain systems (Buehler et al., 2008; Davis et al., 2008). Therefore, evaluating the influence of capture, handling and stress on multiple immune parameters is crucial to reliably study the modulations of immunity in free-living vertebrates. For example, lysis (reflecting complement activity) and agglutination (reflecting natural antibodies) showed no change to handling stress for up to 150 min in Red Knots *Calirdis canutus* (Buehler et al., 2008) and lysis increased 25 min after capture in only one out of five passerine species (Zylberberg, 2015). Similarly, agglutination increased 15 min post capture in only one of these five passerine species (Zylberberg, 2015) but showed no change in the other four; however, house sparrows *Passer domesticus* showed a decrease after 120 min (Gao et al., 2017).

Other studies reported reduced microbicidal activity in response to handling stress in five tropical bird species and brown-headed cowbirds *Molothrus ater* (Matson et al., 2006; Merrill et al., 2012) and a reduction in lysozyme activity (Zylberberg, 2015), decreased natural antibody and complement-mediated activity in the Abert's Towhee *Melozone aberti* (Davies et al., 2016), and reduced cutaneous immune activity in response to corticosterone in sparrows *Passer domesticus* (Martin et al., 2005). However, stress has also been observed to enhance immune parameters. For example, concentrations of haptoglobin, an acute phase protein, increased after 35 min (but not earlier) in one out of the five passerine species, but showed no effect to handling stress in the other species for up to 40 min (Zylberberg, 2015) and phagocytic activity in chickens (Millet et al., 2007). These inconsistent findings may result from most studies using only one measure of immunity as well as sampling at only one point during the stress response. Additionally, life-history and season may influence these results due to the different scales and pressures influencing trade-offs. For instance it has been suggested that there are differences in immune responses to handling stress between tropical and temperate birds (Buehler et al., 2008) as well as experimental evidence for seasonal modulation of sickness behaviour (Owen-Ashley and Wingfield, 2006).

In birds, most empirical studies have been carried out in short-lived passerines or charadriiforms, both in the wild and in captivity. Few studies have focused on long-lived species, which may exist under different stress-immune trade-off balances due to their different life-histories and paces-of-life (Previtali et al., 2012; Tieleman, 2018; Tieleman et al., 2005). However, knowledge of species with different life-histories is crucial if one wants to collect parameters of immune function (or any other physiological trait) unbiased by stress in any evolutionary or ecological study.

Here we investigate the impact of capture on innate constitutive immune function in a long-lived Antarctic seabird the Adélie penguin (*Pygoscelis adeliae*). Adélie penguins can live up to 20 years old (Ainley and deLeiris, 2002) and have a unique physiology with their breeding season involving protracted fasting periods interspersed with intense foraging (Ainley and deLeiris, 2002; Chappell et al., 1993; Vleck and Vleck, 2002). This may cause shifts in energy prioritisation and thus result in different trade-offs between immunity and stress compared to species for which effects of handlings stress on immune function have been evaluated (Vleck and Vleck, 2002). Furthermore, extensive diving in Antarctic marine habitats, meaning they are exposed to a very different set of pathogens compared to terrestrial based temperate species, may affect trade-offs involving immune function. Using three different immune markers (haptoglobin, agglutination and lysis) that are frequently used in ecological and evolutionary studies, we assess the responses of immune markers to holding time in this long-lived Antarctic seabird.

## RESULTS AND DISCUSSION

**Fig. 1.**
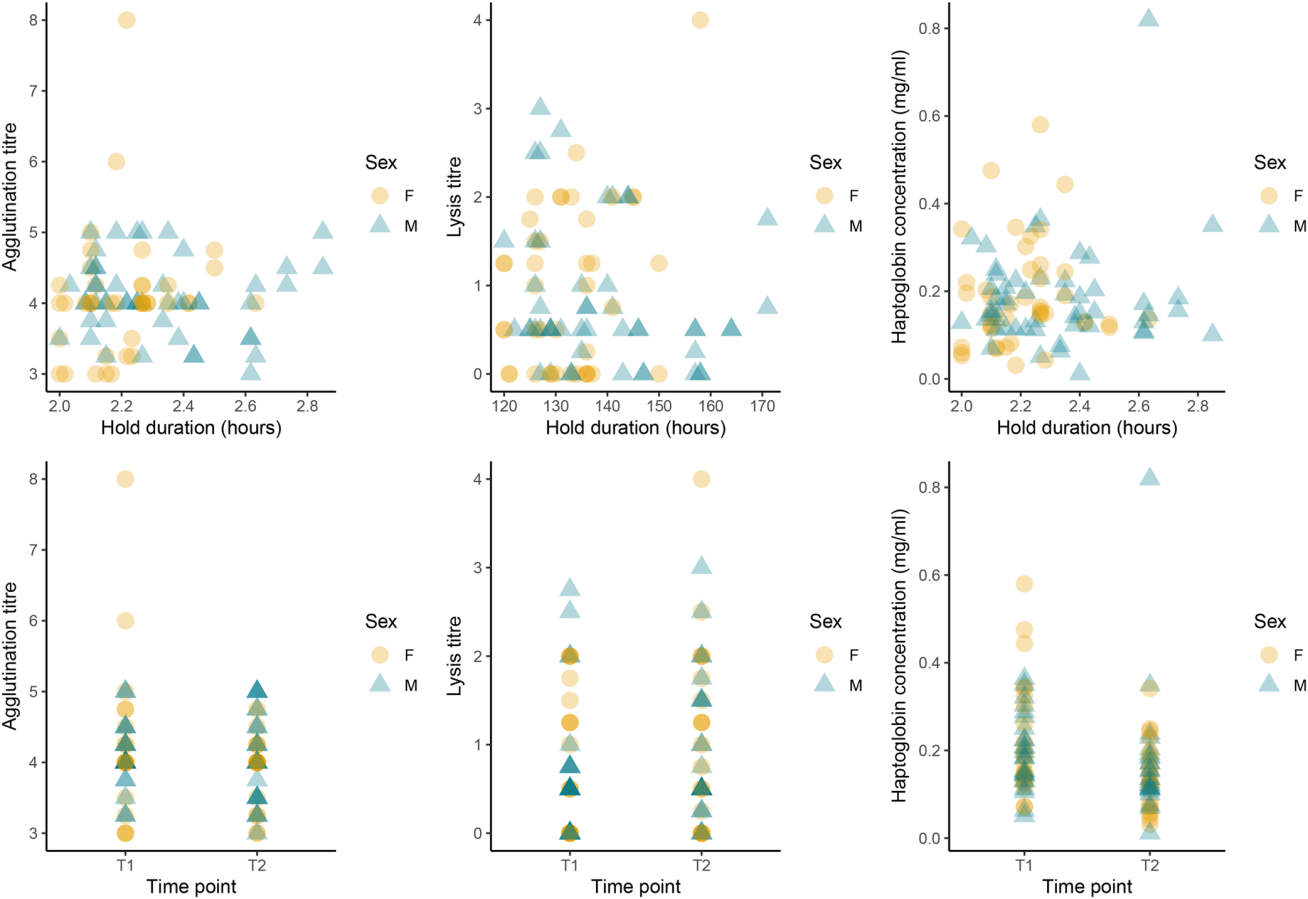
**Relationship between hold duration and time point with respect to three immune function markers, agglutination titer, lysis titer and haptoglobin concentration, in Adélie penguins.** Data points represent individuals and intensity of data points indicate overlapping datapoints. (*N* =58, females represented by yellow circles *n*=24, males represented by blue triangles *n*=34) Neither hold duration or blood sample time point were included in the best supported models of any of the immune markers.

For all three immune parameters (agglutination, lysis and haptoglobin), the null model was best supported and no other models were within 2 ΔAICc of the top model. Though in all cases the second-best supported model contained the term timepoint still within 3 ΔAICc (see Table 1). See Fig. 1 for description of the data distribution and the raw data with respect to the main variables of interest (hold duration and time point). Neither sex nor body mass index (BMI) influenced immune function in any of the three immune marker models.

**Table 1.**
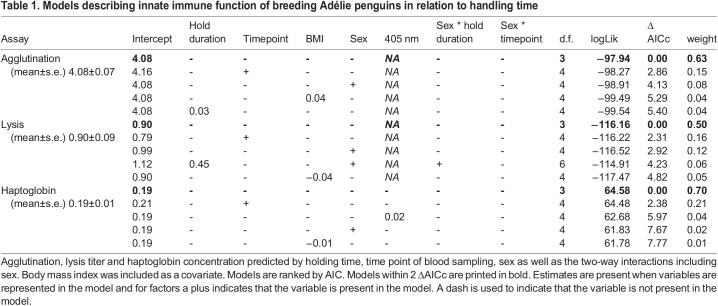
Models describing innate immune function of breeding Adélie penguins in relation to handling time

We investigated the response of three commonly used measures of innate constitutive immune function to holding time in free-living individuals of a long-lived Antarctic seabird. We found that neither agglutination (natural antibodies), lysis (complement activity) or haptoglobin concentration were significantly related to the time point at which the blood sample was taken (before or after captivity time). However, there is evidence for a weak effect of time point given it is present as a term in the second-best supported models. We also found none of the immunity measures to be related to the holding time between samples, suggesting that handling and then holding for up to 2 h does not influence these three parameters of innate constitutive immune function in this species. That there is no impact of time in the best supported model (across a period of about 2 h) is an important finding for those interested in applying measuring of immune function in free-living penguins and potentially other long-lived seabirds, as few studies have focused on handling and immunity in long lived species.

Generally, animals engage stress responses to maximise fitness prospects in their current environment (Angelier and Wingfield, 2013; Wingfield et al., 1998), and changes in immune function as a result of acute stress represent a redistribution of resources in order to optimise the likelihood of the individual to survive the stressor (even if it involves a reduced immunity) (Braude et al., 1999). Though we did not measure any direct parameter of stress (like CORT), our results are in line with those of Buehler et al. (2008) where red knots showed an increase in CORT in response to handling stress, but no change in ten measures of constitutive immune markers, though at shorter time scales (20-30 min from handling). Conversely, however, in short-lived captive house sparrows (*Passer domesticus*), Gao et al. (2017) found agglutination, lysis and microbiocidal activity to change in response to handling stress within 10 min and agglutination to decrease within 120 min. Likewise, Zylberberg (2015) found that some parameters of constitutive innate immune function were sensitive to short-term (within 30 min) handling stress in some passerine species (but not in others). Combined, these results, including our own, might suggest that in long-lived species such as waders and penguins, changes in immune function as a consequence of handling may occur at a slower pace than in short-lived species. However, these differences could be due to other factors, including for example annual-cycle stage at time of sampling, sampling timelines, and/or differences in magnitude of stress response to handling protocols. Furthermore, we cannot rule out that changes occurred in other immune parameters that we did not measure. A phylogenetically controlled meta-analysis could reveal if there is indeed an influence of lifespan on how (fast) immune parameters change upon handling (or other stress).

Though sex differences in immune function during the breeding season are possible, due to the immunomodulating effect of sex hormones, (Owen et al., 2010; Roved et al., 2017) these differences vary between seasons (Valdebenito et al., 2021), and we found no sex differences in any of the three markers of innate immune function measured. Given the monogamous mating system in Adélie penguins where both adults provide care to young this could be expected due to similar levels of investment in reproduction (Hasselquist, 2007). These findings may not remain consistent across all annual-cycle stages for both sexes (Valdebenito et al., 2021).

It is important to reduce human presence, restraint and handling duration as much as possible when working with wild animals in an attempt to minimise stress imposed on the study animals. However, in terms of the methodological insight for future immunology studies, our results indicate that Adélie penguins show a limited impact of handling on three parameters of constitutive innate immune function. Thus samples previously deemed to be unviable for immunological studies may in fact be used for these three immune measures, or experimental protocols incorporating multiple blood samples across time could now be possible without the impact of handling on immunological markers. The type of restraint was mild as the focal penguins were held with a conspecific and outside so that they could see and hear the colony; however, duration was relatively long (up to 2.7 h). There are very few studies of this kind in long lived species such as seabirds, this opens up many possibilities to measure immune function in penguins even when samples cannot be taken within a few minutes of capture. Although birds should be sampled as soon as possible after capture to limit bias, studies of certain baseline immune function in species not easily sampled within a few minutes could still yield unbiased results. Yet we urge researchers to validate this for their own study species, handling protocols, and immune measures of interest.

## MATERIALS AND METHODS

### Data collection procedure

This study was conducted with free-living Adélie penguins during the guard stage of chick rearing when adults alternate between taking foraging trips or guarding the young (21st December 2018-14th January 2019) at Dumont d'Urville station, Terre Adélie, East Antarctica (66°40′S; 140°01′E). We captured 58 adults (24 females and 34 males) on the nest, when both adults were attending the nest prior to a changeover. Only one member of a pair was ever sampled, to reduce disturbance time to the nest.

This study took advantage of a blood sample protocol required for a doubly labelled water (DLW) experiment that necessitated two initial blood samples, one taken after capture and a second one taken after a calibration period in which the animals must be held in captivity (Hicks et al., 2020). Specifically, upon capture, individuals were blood sampled within 3 min of first handling from the tarsus vein. This sample served as a background sample providing the unstressed situation. After blood sampling, birds were weighed to the nearest gram, flipper measured to the nearest millimetre, and injected with 0.3 ml of DLW per kg of body weight into the pectoral muscle [see Hicks et al. (2020) for details]. For future identification, birds were marked with a unique identifying code printed on a piece of marine tape rolled around their back feathers. This entire handling period took about 15 min (study mean) and several previous studies have shown that 15 min of handling evokes a stress response as measured by increases in corticosterone in this species (Cockrem, 2013; Cockrem et al., 2006, 2008) and also in this population (Marciau et al. under review). After this handling period, birds were placed in a contained area outside the lab for the DLW to equilibrate (for between 1.6 and 2.7 h). The contained area measured 2×2.80×5.20 m, birds were always placed in the contained area with another penguin to calm them and the space was filled with a layer of snow for comfort and cooling. A second blood sample was taken after the equilibrium period. This sample served as our handling time sample. Hold duration was calculated as the time between the first capture and the second blood sample. Whole blood was kept on ice in Eppendorf tubes for up to 10 min before being centrifuged (10,000 rpm, 10 min) and plasma stored at −80°C until analysed. The molecular sexing of all individuals was carried out at the service Analyses Biologiques of the Centre d'Etudes Biologiques de Chizé (CEBC). DNA extraction was conducted with 2 µl of pellet (red blood cells) and using a chelex resin (Chelex 100 Molecular Biology Resin, Bio-Rad; 10%) associated with Proteinase K (PK) as written in the manufacturer's instructions. We then performed a polymerase chain reaction (PCR) with amplification of the CHD gene following a standard procedure validated on penguins (Lee et al., 2010).

### Ethics

Comité d'Ethique en Expérimentation Animale Numéro 084, Terres Australes et Antarctiques Françaises, Comité d'Environnement Polaire and Conseil National de la Protection de la Nature all approved this experiment to be carried out and all experiments were performed in accordance with these guidelines and regulations as well as the SCAR Code of Conduct for the use of Animals for scientific purposes in Antarctica. https://www.scar.org/scar-library/search/policy/codes-of-conduct/3408-code-of-conduct-for-the-use-of-animals-for-scientific-purposes-in-antarctica/.

### Immune assays

We used two assays that are commonly and widely used in many ecological and evolutionary studies that assess innate immune function in wild animals (Boughton et al., 2011; Hegemann et al., 2017; Matson et al., 2012). Specifically, we used the haemolysis and haemagglutination assay and quantified haptoglobin concentrations. Haemolysis and haemagglutination assay quantifies natural antibody titers (measured as agglutination titer) and complement activity (i.e. pathogen destruction measured as lysis titer) (Matson et al., 2005). Haptoglobin is an acute phase protein and its concentration often signifies the onset of a non-specific immune response, but concentration changes might also reflect the role of haptoglobin as an antioxidant (Matson et al., 2012) and it also has an association with energetically-expensive systemic inflammation (Buehler et al., 2009; Van De Crommenacker et al., 2010).

All assays were performed blind with respect to time point (i.e. initial capture blood sample or sample after containment) and bird ID. Repeated samples from the same individual were always next to each other on the same plate, otherwise samples were randomised (with respect to sex and Julian date). Owing to plasma limitations, sample size varies slightly among assays (haptoglobin: *n*=51, hemolysis-haemagglutination: *n*=49). Samples were refrozen between the two assays, as those assays are robust against multiple freeze-thaw cycles (Hegemann et al., 2017).

### Haemolysis and haemagglutination

We followed the protocol outlined by Matson et al. (2005) for hemolysis and hemagglutination assay. Red blood cells (RBC) from rabbits (Envigo RMS Ltd, UK) were incubated in serially diluted penguin plasma samples. Agglutination and lysis were recorded as titers (−log2 of the last plasma dilution that shows each reaction). Agglutination was scored (1-9) from assay plate images recorded 20 min after incubation, and lysis was scored from images recorded 90 min after incubation. Blind to sample and plate identity, O.H. scored randomised images from each time point a minimum of two times. If the first two scores were <1 titer apart, then we used the mean value in further analyses. If these first two scores were ≥1 titer apart, then the sample was scored a third time, and we used the median value in further analyses. The average within-plate variation (standard deviation) was 0.75 lysis titres and 0.57 agglutination titres. The average among-plates variation was 0.46 lysis titres and 0.37 agglutination titres.

### Haptoglobin

We quantified haptoglobin concentration ([Hp] (mg mL^−1^)) from the undiluted plasma undiluted plasma samples in singlet using a commercially available functional assay (TP801; Tri-Delta Diagnostics, NJ, USA). We followed the methods provided by the manufacturer with a few minor modifications.

We measured absorbances at three wavelengths (405, 450 and 630 nm) prior to the addition of the final reagent that initiated the colour change reaction. The 405 and 450 nm pre-scan enabled us to statistically analyse and correct for differences in plasma sample redness, an indication of hemolysis, which can affect the assay (Matson et al., 2012; Wemer et al., 2021).

### Statistical analyses

Mixed effects models were run separately for each immune marker in statistical software version 3.6.2 R. We modelled immune marker as a function of blood sampling time point and hold duration. We included sex as a covariate because in some species immune function differs between males and females (Valdebenito et al., 2021). We also included a body mass index (body mass/flipper length) (Cockrem et al., 2006) due to potential correlation with parameters of immune function (Hegemann et al., 2012). We also included interaction terms between sampling point and sex and hold duration and sex. Bird ID was fitted as a random effect to control for multiple samples per bird. When modelling haptoglobin concentration, 405 nm wavelength was included as a covariate to control for plasma redness as this explained more variation than the 450 nm measurement. For all models, numerical predictor variables were scaled and centred to allow for direct comparison of estimates. Models were evaluated using an information theoretic approach (Burnham and Anderson, 2001) and models were ranked using sample size corrected Akaike Information Criterion (AICc), with the best model having the lowest AICc value. We calculated ΔAICc as the difference in AICc between each candidate model and the model with the lowest AICc value and considered all models within 2 ΔAICc as competitive models (Burnham and Anderson, 2001).

## Supplementary Material

10.1242/biolopen.059512_sup1 Supplementary information
